# Metaheuristic-Based Feature Selection Methods for Diagnosing Sarcopenia with Machine Learning Algorithms

**DOI:** 10.3390/biomimetics9030179

**Published:** 2024-03-15

**Authors:** Jaehyeong Lee, Yourim Yoon, Jiyoun Kim, Yong-Hyuk Kim

**Affiliations:** 1 Department of IT Convergence, Gachon University, 1342 Seongnamdaero, Sujeong-gu, Seongnam-si 13120, Gyeonggi-do, Republic of Korea; ljh365365@gachon.ac.kr; 2 Department of Computer Engineering, Gachon University, 1342 Seongnamdaero, Sujeong-gu, Seongnam-si 13120, Gyeonggi-do, Republic of Korea; 3 Department of Exercise Rehabilitation, Gachon University, 191 Hambakmoe-ro, Yeonsu-gu, Incheon 21936, Republic of Korea; eve14jiyoun@gachon.ac.kr; 4 School of Software, Kwangwoon University, 20 Kwangwoon-ro, Nowon-gu, Seoul 01897, Republic of Korea; yhdfly@kw.ac.kr

**Keywords:** metaheuristic, feature selection, genetic algorithms, harmony search, machine learning, sarcopenia

## Abstract

This study explores the efficacy of metaheuristic-based feature selection in improving machine learning performance for diagnosing sarcopenia. Extraction and utilization of features significantly impacting diagnosis efficacy emerge as a critical facet when applying machine learning for sarcopenia diagnosis. Using data from the 8th Korean Longitudinal Study on Aging (KLoSA), this study examines harmony search (HS) and the genetic algorithm (GA) for feature selection. Evaluation of the resulting feature set involves a decision tree, a random forest, a support vector machine, and naïve bayes algorithms. As a result, the HS-derived feature set trained with a support vector machine yielded an accuracy of 0.785 and a weighted F1 score of 0.782, which outperformed traditional methods. These findings underscore the competitive edge of metaheuristic-based selection, demonstrating its potential in advancing sarcopenia diagnosis. This study advocates for further exploration of metaheuristic-based feature selection’s pivotal role in future sarcopenia research.

## 1. Introduction

### 1.1. Background and Purpose of Study

Sarcopenia, a geriatric condition characterized by diminished skeletal muscle mass and function, poses heightened risks of falls, fractures, and physical impairment. Prevalence of the condition increases notably among individuals in their 80s compared to those in their 60s and 70s. This suggests a potential rise in affected individuals, coinciding with increased life expectancy. Consequently, addressing sarcopenia has become imperative as we navigate an era dominated by aging demographics. However, despite its significance, the precise mechanisms underlying sarcopenia remain elusive, confounding attempts to pinpoint causative factors [1,2,3].

Efforts to tackle this challenge have led to studies employing machine learning methodologies in sarcopenia diagnosis, offering novel insights beyond human capacity. Some methodologies integrate diverse datasets, such as microarrays, surveys, or imaging data, to discern crucial patient features for diagnosis [3,4,5]. For instance, Kang et al. [5] utilized a random forest algorithm to select features based on various clinical criteria. The study acknowledges a limitation in feature selection, which is heavily dependent on expert knowledge. This prompts exploration into the possibility of computer algorithms autonomously identifying diagnostic features. However, the escalating number of features presents challenges in identifying the optimal feature set, necessitating the implementation of algorithms capable of robust performance in selection.

This study aims to experimentally validate the potential of employing a metaheuristic algorithm for sarcopenia diagnosis through feature selection. Leveraging survey-based data, we conducted experiments using the genetic algorithm and harmony search, among other metaheuristic approaches, to identify suitable feature sets. Comparative analysis against conventional algorithms shed light on the efficacy of metaheuristic-based approaches in machine learning for sarcopenia diagnosis.

The remainder of this paper is organized as follows. In the rest of Section 1, we review related work and introduce the contributions of our work. In Section 2, we explain the proposed method in this study, followed by the experimental setup process in Section 3 and the results in Section 4. Section 4 also includes a summarization and discussion of our results. Finally, we conclude with contributions and limitations of the work in Section 5.

### 1.2. Related Work and Contributions of the Study

While sarcopenia’s recognition as a disease is relatively recent, researchers have diligently pursued its diagnosis and the identification of contributing factors. Numerous studies have strived to establish diagnostic criteria and employ diverse methods—from exercise testing to IoT-enabled body measurements—to pinpoint sarcopenia [6]. Survey-based investigations, such as those using data from the National Health and Nutrition Examination Surveys (NHANES) by the Centers for Disease Control and Prevention (CDC), revealed potential links between sarcopenia susceptibility and inflammatory diets and non-alcoholic fatty liver [7,8]. Past surveys heavily relied on statistical techniques such as correlation and feature analysis to unravel sarcopenia-associated factors.

Machine learning has emerged as a potent tool for enhancing sarcopenia definition and diagnosis, uncovering patterns beyond human observation, and gaining deeper insights [9,10]. Ongoing research explores machine learning’s role in diagnosing and understanding sarcopenia and promising results have been achieved using various datasets and methodologies [11,12]. Some studies utilize machine learning to unveil sarcopenia factors and predict their occurrence from clinical records [5,13]. Researchers additionally explore cutting-edge technologies such as MRI and CT imaging, deep learning, or reinforcement learning techniques for sarcopenia diagnosis. Innovative systems that integrate cloud computing have also been investigated [3,14,15].

However, limitations for traditional studies, particularly in machine learning methodologies, are being debated. The issues include limited data access, imbalanced data, and feature selection complexities that pose challenges, especially with excess features impacting diagnostic accuracy [6,9]. Furthermore, related work also has certain limitations. As prior survey-based investigations predict sarcopenia using statistical techniques, the methods necessitate intricate calculations and rules for identifying diagnostic criteria or factors. The studies applying machine learning to diagnose sarcopenia and identify crucial factors are constrained by the requirement of human intuition and domain-specific knowledge data. Consequently, all preceding research is restricted by the potential for direct human intervention in judgment. This prompts whether machines can autonomously diagnose sarcopenia without human assistance and screen for its contributing factors.

This study aims to enhance feature selection by introducing metaheuristics, specifically harmony search and the genetic algorithm, to bolster sarcopenia diagnosis performance. The experimentation distinguishes itself by addressing previous concerns through metaheuristic applications. It highlights the effectiveness of these methods in sarcopenia diagnosis and provides valuable insights for future research considerations. Indeed, this study holds significance for introducing a metaheuristic approach to sarcopenia research and discussing its effectiveness and limitations. Through a series of experiments, we demonstrate the application of metaheuristics into machine learning for sarcopenia diagnosis and assess their performance relative to other algorithms. Furthermore, our work contributes to the field by indicating that algorithms can enhance sarcopenia diagnosis performance independent of human knowledge domains and facilitate factor screening.

## 2. Materials and Methods

### 2.1. Diagnosing Sarcopenia

The development of sarcopenia involves a complex interplay influenced by diverse factors such as age, gender, race, and environment. Ongoing comprehensive research is necessary to fully comprehend it and establish clear diagnostic criteria [6]. Both the Asian Working Group for Sarcopenia (AWGS) and the European Working Group for Sarcopenia (EWGS) have pinpointed low muscle mass, strength, and physical activity capacity as critical diagnostic criteria and have proposed guidelines accordingly [6,16,17].

Diagnostic tools such as SARC-F incorporate muscle strength, assisted walking, rising from a chair, stair climbing, and falls to diagnose sarcopenia [18]. Other measurements such as gait speed [19] and the Timed Up and Go (TUG) test [20], not covered by SARC-F, have also been utilized due to their established link with sarcopenia. While some studies use a combination of metrics such as SARC-F, others independently leverage specific metrics for diagnosing sarcopenia [16,18,20]. Additionally, studies have explored indirect prediction of sarcopenia through patient data [5,11,12], often employing feature selection to identify causative factors, yielding varying results in features and accuracy [21,22].

Direct diagnosis through muscle strength remains a prominent approach, providing a convenient and easily applicable metric in diverse clinical scenarios, particularly for early-stage sarcopenia detection [6,16]. Diagnosing sarcopenia with muscle strength challenges such as differing diagnostic criteria among ethnicities and regions highlights the need for standardized measurement methods [6,23,24]. However, measuring muscle strength, particularly handgrip strength, retains its validity as a diagnostic criterion. This is due to its simplicity, convenience, and consistency in delineating sarcopenia diagnostic criteria, including upper body function [23,25]. In particular, Verstraeten et al. [26] explain that handgrip strength may be a more useful diagnostic indicator than the rising from a chair test because of its ability to predict adverse outcomes. Therefore, we decided to diagnose sarcopenia using handgrip strength in this study.

### 2.2. Feature Selection

Feature selection in machine learning involves selecting pertinent features related to predicted data and utilizing them for learning. Unlike feature extraction, which generates a new set of relatively low-dimensional features from existing ones, feature selection specifically picks and incorporates only the necessary features for learning. Thereby, feature selection filters out relatively irrelevant features, presenting an advantage [27].

Guyon and Elisseeff [28] highlight numerous benefits of feature selection in machine learning. They explain these benefits, which include enhancing data visualization and understanding, reducing computational complexity, and mitigating the curse of dimensionality. This eliminates unnecessary features in high-dimensional data, proving its significance in successful machine learning applications [27,29].

Feature selection algorithms fall into three broad categories: filter method, wrapper method, and embedded method. The filter method evaluates each feature independently using the predicted label, while the wrapper method employs a predefined algorithm’s performance to assess selected features. The embedded method integrates feature selection during machine learning model training, acting as a middle ground between the two methods [28,29]. Although computationally less intensive, the filter method might yield suboptimal solutions for machine learning. In contrast, the wrapper method’s disadvantage lies in its extensive search space, which could be more problematic in high-dimensional datasets. Conversely, low-dimensional datasets mitigate this disadvantage due to smaller search spaces [29].

Solving the feature selection problem exhaustively by inspecting all feature sets is impractical given the exponential growth in possible subsets with the number of features. An optimal solution through exploring all possible feature sets is infeasible due to its exponential complexity [30,31]. Hence, employing metaheuristic-based algorithms becomes pertinent as they explore a subset of the solution space to identify the optimal feature set, avoiding exhaustive exploration. These algorithms, combined with optimization methods, efficiently find high-quality solutions with minimal computational efforts. This emphasizes the potential of metaheuristic-based feature selection [30,32,33]. Therefore, this study opts for a metaheuristic-based algorithm for feature selection.

### 2.3. The Genetic Algorithm

The genetic algorithm (GA), initially conceptualized by Holland in 1975 as a mimicry of biological evolution, simulates the evolution of individuals to discover optimized solutions. Similarly to the combination and mutation of genes in living organisms, these algorithms utilize crossover and mutation operations to generate new individuals, gradually converging toward a specific solution over generations [34].

However, genetic algorithms require a definitive overarching rule, which leads to no assurance of finding the required answer. The nature of convergence might limit the solution to a suboptimal result without guaranteeing the best solution [35]. Despite this, their efficacy in exploring vast search spaces and tackling complex problems, notably NP-hard problems such as feature selection, makes them a fitting choice. This is due to their capability to navigate challenging problems and potentially discover optimal combinations [36,37]. Hence, adopting genetic algorithms aligns with the goals of this study.

**Figure 1:**
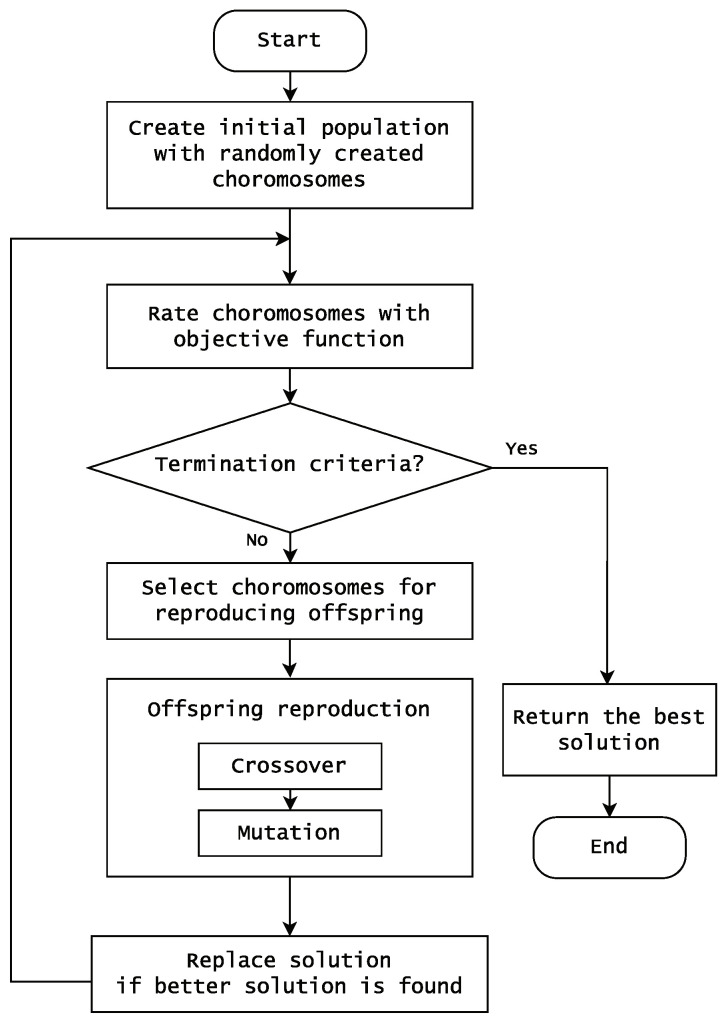
A flowchart for a typical genetic algorithm.

Typically, genetic algorithms commence with a randomly generated population. Choromosomes are assessed and assigned probabilities for transmission to the next generation based on their alignment with problem criteria. The selected choromosomes undergo recombination in various ways, generating new choromosomes that meet specific criteria. Occasionally, a mutation operation alters the recombined solution with a certain probability, and this iterative process continues until the algorithm’s termination condition is met [34,38]. Figure 1 illustrates a flowchart depicting this process.

Essential points in genetic algorithms are crossover and mutation operations. A crossover enables the generation of offspring solutions by exchanging components of parent solutions, while mutation explores previously unexplored or overlooked solution spaces. These operations significantly influence the algorithm’s ability to maintain diversity within the population and its capacity to converge toward optimal solutions [39].

### 2.4. Harmony Search

The harmony search (HS) algorithm, conceived by Geem et al. [40], draws inspiration from musical improvisation. It stands out as a potent metaheuristic method for solving optimization problems, rivaling traditional metaheuristics in efficacy [41]. The algorithm employs memory consideration and pitch adjustment, enabling efficient solution space exploration while retaining proximity to promising solutions [42].

**Figure 2:**
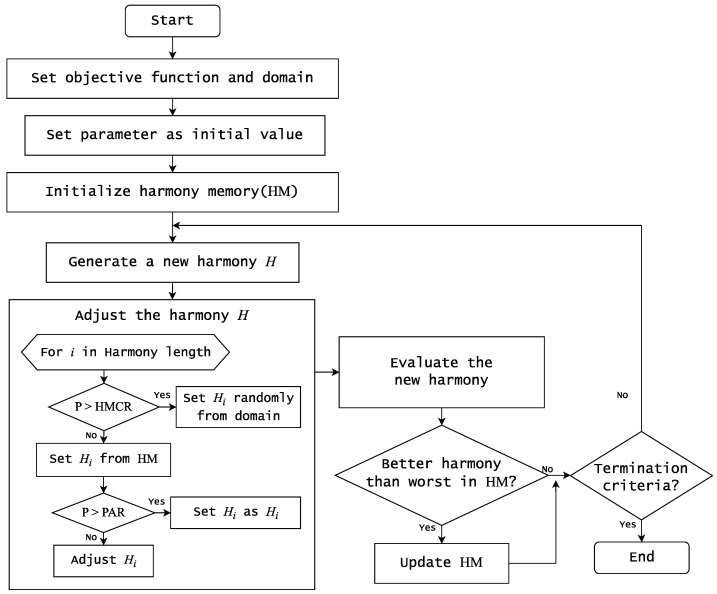
A flowchart for typical harmony search.

The operational principle of harmony search involves the following steps [32,40,41], as illustrated by the flowchart in Figure 2:

1. Initialization of the problem and its parameters;
2. Random initialization of harmonies in the solution set;
3. Generation and evaluation of new harmonies;
4. Replacement of existing worst harmonies with newly created ones if they yield better fitness;
5. Reiteration of Steps 3 and 4 until meeting termination conditions.

The critical parameters of harmony search include HMS (harmony memory size), HMCR (harmony memory consideration rate), and PAR (pitch adjustment rate). HMS determines the number of solution sets, while HMCR influences the referencing of previously created values when generating new solutions. A higher HMCR increases the likelihood of referencing stored values, while a lower value favors random selection. PAR dictates the adjustment probability of fetched solutions, with a higher value indicating a greater likelihood of adjustment and use. The settings of these parameters significantly impact solution quality due to the algorithm’s probabilistic nature [40,41,43].

However, fixed parameter values, as proposed by Geem et al. [40], can prolong the optimization process, potentially necessitating further optimization to identify optimal settings [32,44]. The absence of general rules for parameter control emphasizes the need to iteratively vary parameters, enhancing the algorithm’s speed and efficiency [43,44]. To solve this obstacle, dynamic parameter tuning is introduced to the original harmony search algorithm to address its limitations. Incorporating dynamic parameter tuning or additional components into the algorithm can mitigate these challenges, which is ultimately expected to enhance solution outcomes [43]. Thus, in this study, we aim to implement parameter dynamic tuning in harmony search and analyze the results.

Moreover, extending this concept of dynamic parameter tuning to genetic algorithms is also part of our investigation. By introducing dynamic parameter tuning to genetic algorithms, we aim to explore its impact and compare the outcomes with those achieved through dynamic tuning with harmony search. This comparative analysis seeks to unveil the efficacy of dynamic parameter tuning in both algorithms and assess their performance enhancements.

## 3. Experiment Setup

### 3.1. Dataset

We utilized the Korean Longitudinal Study of Aging (KLoSA) dataset (retrieved from https://survey.keis.or.kr/eng/klosa/klosa01.jsp, accessed on 3 November 2023). The dataset that was used to diagnose sarcopenia was sourced from the Korea Employment Information Service, specifically the 8th KLoSA survey in 2020, and was the latest available as of 2023. This survey encompassed middle-aged and older individuals born before 1962, residing in South Korea (excluding Jeju Island), and involved around 10,000 respondents. Survey components spanned various domains: demographics, family ties, health status, employment, income, consumption, assets, subjective expectations, and quality of life.

**Figure 3:**
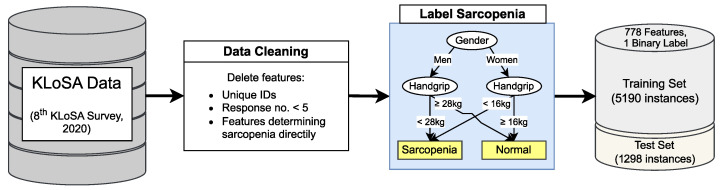
An overview of dataset preparation and preprocessing.

To streamline our analysis, we conducted data preprocessing on the KLoSA dataset. Initially, we excluded unreliable features such as unique IDs and items with limited responses (five or fewer). Given sarcopenia’s multifaceted nature and elusive etiology [2,3,4], we let algorithms consider comprehensive coverage across the KLoSA survey categories while selecting features. This approach aimed to uncover novel factors influencing sarcopenia diagnosis and shed light on the interplay between multiple factors and sarcopenia. Subsequently, adhering to Korean diagnostic criteria [16,45], individuals with handgrip strength below 28 kg for men and 16 kg for women were diagnosed with sarcopenia. Undiagnostic data, especially missing hand grip data, were then removed. Features directly related to sarcopenia determination, such as handgrip strength data, were also excluded. Further, we partitioned the dataset: 80% was used as a training set and 20% was used as a test set, ensuring an even distribution of diagnosis results. After preprocessing, we established 778 features and a single binary label across 5190 training and 1298 test instances. Furthermore, the distribution of sarcopenia and normality is shown in Table 1. The preprocessing process is illustrated in Figure 3.

However, we opted not to apply separate feature engineering techniques in our study. Indeed, it is crucial to deal with feature engineering problems in machine learning and data science. Survey data present a diverse mix of data types, posing challenges for feature engineering. For instance, numeric data may contain outliers necessitating standardization, while other data may be presented in ranges, making normalization more appropriate. Effectively handling these various data types can be complex and, if performed incorrectly, may hinder machine learning performance. Additionally, the computational nature of NaiveBayes, which we utilized as a wrapper, allows for robust machine learning without extensive feature engineering. Consequently, the impact of not applying feature engineering in our feature selection process was minimal. For the same reason, we did not perform any particular missing-value corrections. Different categories of data require different ways to handle missing values, and different machine learning algorithms need particular and effective ways of handling data imputation. If the process mishandled data, machine learning could be interfered. The WEKA framework we used considers this and handles it automatically, so we did not perform any additional missing-value corrections [46].

### 3.2. The Metaheuristic Algorithm

#### 3.2.1. Algorithm Implementation

Genetic algorithm implementation for our experiments followed Lee et al.’s method [22] as shown in Figure 1. We introduced binary encoding to represent solutions, assigning a length equivalent to the number of features in the dataset to each solution. Within this encoding, a selected feature was denoted by 1, while an unselected one was marked as 0. Initially, the population comprised 30 solutions. Conversely to requiring a fixed number of features for initial solutions, the number of randomly chosen solutions was determined individually for each generation.

Assessment of generated solutions utilized the naive Bayes classifier (NaiveBayes) within WEKA [46]. The solution achieving the highest accuracy via 5-fold cross-validation was deemed the best solution. Furthermore, we evenly distributed the data during cross-validation to maintain a balanced label ratio. By applying cross-validation to the wrapper that evaluated the solution from a metaheuristic, we could provide a more general evaluation of the solutions found by the algorithm. This approach also guards against overfitting during feature selection and guarantees an impartial evaluation of the solutions. NaiveBayes, chosen as a wrapper, proved suitable due to its swift performance and effectiveness in real-world classification tasks [47]. We used roulette-wheel selection for the selection mechanism. We designed it so that only half of the solutions survived and were replaced by new ones in each generation, with the top three solutions persisting unconditionally. Employing one-point crossover and bit-flip mutation methods, the genetic algorithm’s parameters were determined and are given in Table 2.

On the other hand, our harmony search implementation followed a fundamental algorithmic sequence outlined in Figure 2. Similar to the genetic algorithm, our solution was represented in binary encoding. The evaluation of our solution relied on accuracy that was assessed via 5-fold cross-validation using the NaiveBayes classifier. The parameters used for our harmony search experiment are summarized in Table 3.

Feature selection is a technique to identify and select relevant features for a specific objective or goal. It can be conceptualized as a binary decision process where each feature is categorized as either ‘selected’ or ‘not selected’. Only those features marked as ‘selected’ are included in the final subset. The resulting feature set is then evaluated against a predetermined metric.

The changes we made to our algorithm to incorporate feature selection into the metaheuristic were mainly binary encoding and the set object function was used as an evaluating wrapper. Binary encoding is a suitable abstraction for representing the selection process within the metaheuristic. This encoding effectively captures the choices made by the algorithm for each feature, offering a straightforward representation compared to other encoding methods. The wrapper method evaluates the solution as performed by a machine learning algorithm. This approach facilitates predicting performance more effectively. Additionally, the performance metrics used for evaluation were intuitive, which made it easy to evaluate which feature set was superior.

#### 3.2.2. Dynamic Parameter Adjustment

Since metaheuristics rely on hyperparameters, setting them appropriately is crucial. However, determining the optimal parameter values for a given problem is challenging due to the absence of universal rules and the multitude of factors involved [43,48]. While optimal parameters are often provided with algorithm proposals, such as in Geem et al. [40], most researchers resort to parameter exploration techniques, such as grid search [49], or develop optimization frameworks to mitigate additional optimization challenges [48] and achieve improved solutions [32].

To enhance those pros in feature selection, we addressed the limitations of the original harmony search algorithm by introducing a dynamic HMCR in this experiment. With this approach, the parameter HMCR is varied across each iteration. This adjustment stems from the algorithm’s working principle, recognizing that, initially, values in the solution set are less likely to be optimal but become more probable with increased iterations. A smaller HMCR facilitates exploring a broader solution space, while a larger HMCR aids fine-tuning and faster convergence [32]. Hence, under a dynamic HMCR, we initially linearly varied the HMCR to explore a broader range of solutions and refined the converged solution later in the process.

Contrarily, we maintained a fixed value without dynamic adjustment for the PAR, another critical parameter. This decision aligned with representing solutions in binary encoding, when tuning with PAR parallels bit-flip mutation in a genetic algorithm. Dynamic changes in PAR could restrict mutation in high-PAR settings, leading to false convergence. Therefore, a fixed PAR value ensures stable solution convergence. In contrast, the genetic algorithm dynamically adjusts mutation probability. Despite the risk of false convergence, dynamic mutations offer a more comprehensive view of solutions in the current framework. As altering crossover parameters has not improved convergence [50], we tuned mutation probability similarly to dynamic HMCR, varying it linearly. However, unlike dynamic HMCR, the mutation rate is initially large and decreases as more generations are created. We also took measures to ensure the probability remained above a certain threshold to prevent premature convergence. By tuning these parameters linearly, we alleviated the metaheuristic from incurring additional computational costs, and, by allowing this process to unfold gradually, we mitigated abrupt changes in the solution [32]. The parameter adjustment ranges are summarized in Table 4.

### 3.3. Feature Set Evaluation

We employed several classification algorithms available in WEKA [46] to assess the feature sets obtained from the feature selection algorithms. Specially, we evaluated the feature sets with a decision tree (J48), a random forest (RandomForest), a support vector machine (SMO), and the naive Bayes classifier (NaiveBayes). Hyperparameters for the machine learning algorithms were set to their default values as provided by WEKA (see Table A1 in Appendix A), and none of the changes were made in the algorithms. To compare experimental outcomes based on the metaheuristics’ iterations, we evaluated feature sets generated by our genetic algorithm across 100, 500, and 1000 generations. For the harmony search, evaluations were conducted across 1000, 5000, and 10,000 iterations, respectively. The genetic algorithm generated half of the solution as new solutions per generation, totaling 1500, 7500, and 15,000 new solutions for the specified generations. Conversely, harmony search produced one solution per iteration.

For benchmarking against traditional feature selection methods in algorithms coupled with metaheuristics, we utilized CfsSubsetEval (CFS) [51] and InfoGainAttributeEval (IG) [52]. These filter-method techniques, implemented in WEKA [46], assess feature sets using correlation coefficients and information gain. Respectively, CfsSubsetEval gauges predictive power and redundancy between features, often combined with greedy algorithms such as BestFirst. Meanwhile, InfoGainAttributeEval measures information gain for feature selection, where higher values signify a stronger correlation with the classification label. These methods focus on feature values rather than black-box approaches, contrasting with the NaiveBayes-driven metaheuristic algorithm used in this study. This choice facilitates a comparison between filter and wrapper methods. Under CfsSubsetEval, we utilized the feature set calculated within the BestFirst algorithm. For InfoGainAttributeEval, we selected features with an information gain of 0.1 or higher.

As depicted in Table 1, the dataset utilized in our study exhibits label imbalance, necessitating a machine learning evaluation metric that accounts for this disparity. Evaluating performance solely based on accuracy fails to address this issue and may yield biased outcomes. To mitigate this, we opted to introduce a weighted F1 score to rectify this challenge that considers data imbalance by calculating an F1 score for each label, which is calculated using Equation (1):

(1) $$F1_{wg}=\frac{\sum (F1_{cls}\cdot N_{cls})}{N_{all}}$$

In classification tasks, the weighted F1 score corresponds to the number of categories into which the target label can be divided. To compute this score, the F1 score is individually calculated for each category ($F1_{cls}$), then multiplied by the number of instances ($N_{cls}$) within that category. These values are then summed across all categories and divided by the total dataset size ($N_{all}$). This process yields a weighted average that considers the data distribution in each category. It results in a more robust F1 score, which is particularly beneficial in scenarios with imbalanced data distributions.

## 4. Experimental Results and Discussion

### 4.1. Comparison with Traditional Feature Selection Methods

**Figure 4:**
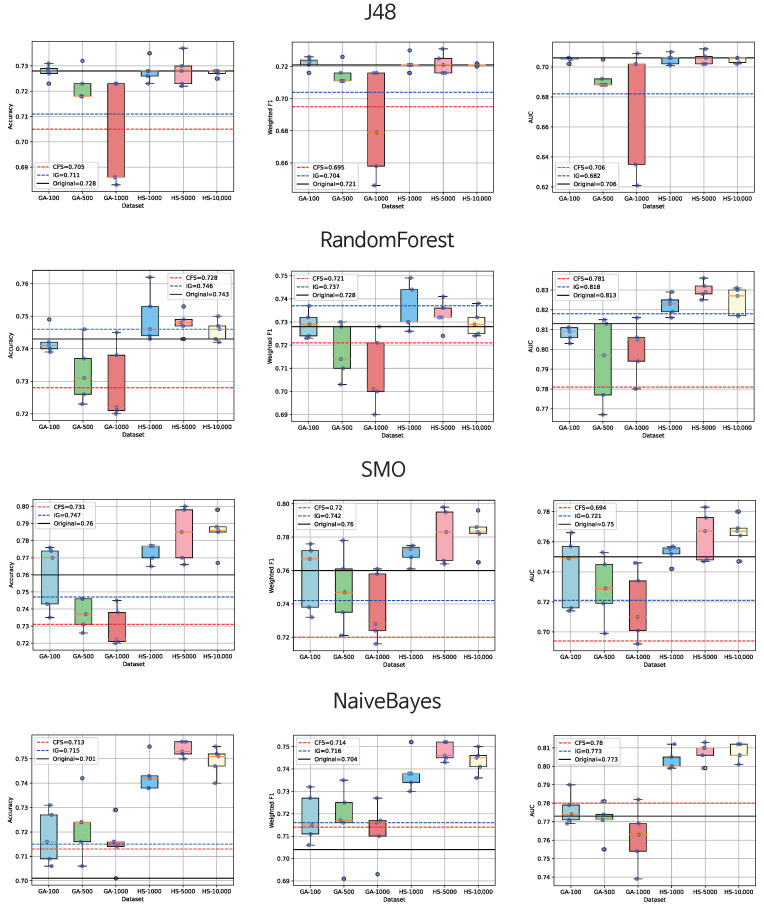
Box plots for machine learning performance on various feature sets.

In our study, we conducted experiments to assess the potential enhancement of sarcopenia diagnosis performance by employing metaheuristics and compared them with established feature selection algorithms. We applied our experiments on a computer with an Intel i7-8750H CPU (Intel, Santa Clara, CA, USA), 16 GB of RAM, and a GTX 1060 mobile GPU (NVIDIA, Santa Clara, CA, USA). The summarized outcomes are presented in Table 5 and Figure 4. Notably, the data showcasing the highest performance for each machine learning algorithm are highlighted in bold for reference. We also summarized feature selection time costs for each algorithm in the table. Additionally, note that all metaheuristic results presented in the tables in Section 4 were obtained by running the same algorithm five times and then averaging the outcomes.

The results given in Table 5 underline a potential for enhanced performance through metaheuristic-based feature selection methods. Except for RandomForest, applying genetic algorithms and harmony search revealed performance improvements across most machine learning algorithms compared with applying traditional methods on average, especially compared with CFS. Notably, the combination yielding the most robust diagnostic performance for accuracy and weighted F1 score was observed when applying SMO on the feature set obtained via harmony search with 10,000 iterations. The highest AUC was achieved with the combination of harmony search with 5000 iterations. Harmony search consistently upheld or enhanced diagnostic accuracy across various scenarios except for J48 with 10,000 iterations. Conversely, traditional methods such as CFS and IG did not exhibit performance improvements, especially CFS, which demonstrated a decline across all the cases except for NaiveBayes. Furthermore, the feature combination from harmony search after 5000 iterations was equivalent to at least or outperformed the original data in all machine learning experiments. These findings suggest that metaheuristic-based feature selection might hold an edge over traditional methods in sarcopenia diagnosis.

Particularly noteworthy were the superior results from the harmony search. Harmony search yielded a more effective solution set despite generating fewer solutions than genetic algorithms. This could be attributed to the algorithm’s computation process. The one-point crossover used in genetic algorithms can falter if not executed at the right point, posing challenges when solutions have good features dispersed throughout [53]. Harmony search, employing value adjustment for all features via HMCR, did not encounter these issues. To address this challenge within the genetic algorithm, employing a broader range of operations in the crossover process—such as multi-point crossover or cycle crossover—could be advantageous. Considering that the data in this study were categorized into bins, leveraging the bin cutoff points as reference markers for the one-point crossover operation might prove beneficial.

However, our experiments also revealed that longer iterations cannot always guarantee superior performance. For instance, in genetic algorithms, the performance of RandomForest and SMO recorded the best results with feature sets obtained with 100 generations. The performance with 1000 generations was inferior to that with 500 generations. Similarly, RandomForest’s results with harmony search showed reduced performance as the number of iterations increased. The 10,000 iteration run performed worse than the original data. This highlights the importance of determining the optimal number of generations for metaheuristic algorithms to outperform traditional ones and the original data. Otherwise, the advantages of metaheuristic feature selection may not manifest. The findings also indicate that NaiveBayes exhibited a comparatively smaller decline in performance compared to other machine learning methods. Despite some degradation in performance, NaiveBayes fared relatively well, particularly in terms of accuracy, which served as the primary criterion for evaluating solutions in the metaheuristic process. While it may have appeared that the NaiveBayes-based classifier performed proficiently, it is reasonable to assume that the wrapper’s reliance on NaiveBayes accuracy may have led to overfitting. To ensure a more balanced evaluation of feature selection, it is imperative to construct a black box with multiple classifiers or employ evaluation criteria that are not reliant on a specific metric.

Additionally, it is important to reconsider whether or not feature sets obtained via metaheuristics are constantly superior. Many studies suggest that optimal feature sets are small and improve machine learning performance [28,54]. However, the sets derived through metaheuristic-based feature selection contained more than 100 features, especially with harmony search, which consistently selected over 200 features. In contrast, traditional feature selection methods selected fewer than 100 features. This indicates that while metaheuristic-based selection yields larger feature sets it might compromise performance. Meanwhile, traditional methods produce smaller feature sets but might not optimize performance. For future metaheuristics to achieve superior performance and small feature sets that are essential for feature selection, additional parameters or constraints might need consideration, which emphasizes that further exploration is needed in this domain.

### 4.2. The Effect of Dynamic Parameter Adjustment

We have extensively explored how fine-tuned parameters impact algorithm performance, as evidenced by the comparative analysis of generational tuning. In the context of parameter manipulation, it is pertinent to examine the effectiveness of dynamic HMCR within the framework of applying harmony search and dynamic mutation rate within the genetic algorithm, respectively.

To investigate this, we initially compared the results of the harmony search employing dynamic HMCR against results obtained with fixed HMCR parameters set at 0.7 and 0.85. Table 6 present these results. Contrary to our expectation, we saw the best performance with the fixed HMCR. Especially, the result demonstrated superior accuracy and weighted F1 score performance in experiments featuring the fixed HMCR. In addition, in the case of J48, the best results were observed when HMCR was fixed, particularly regarding the weighted F1 score. The overall superior performance was generally acheived when the HMCR was fixed to 0.85 for SMO and NaiveBayes and 0.7 for J48 and RandomForest.

On the other hand, the results of the dynamic mutation rate, as given in Table 7, showed the highest scores. However, it would be premature to infer that dynamically adjusting the mutation rate consistently enhances performance. Table 7 illustrates that dynamic parameter tuning may not consistently enhance performance across all algorithms. Specifically, NaiveBayes achieved its best performance when the mutation rate was fixed at 0.1. These experiments suggest that dynamically tuned parameters possibily yield performance enhancements. However, it is crucial to note that they do not always guarantee a performance booster; a misguided application may result in losses without commensurate performance gains.

**Figure 5:**
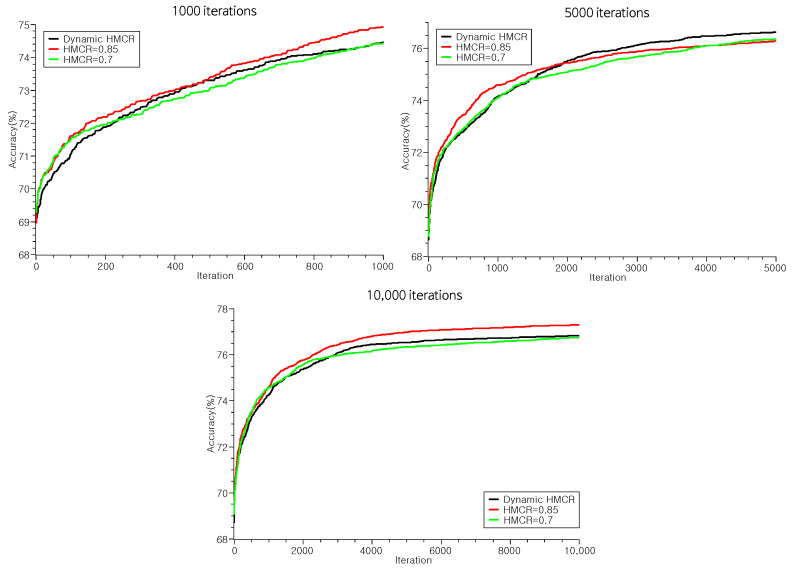
The average accuracy of the solutions founded by our harmony search according to HMCR.

**Figure 6:**
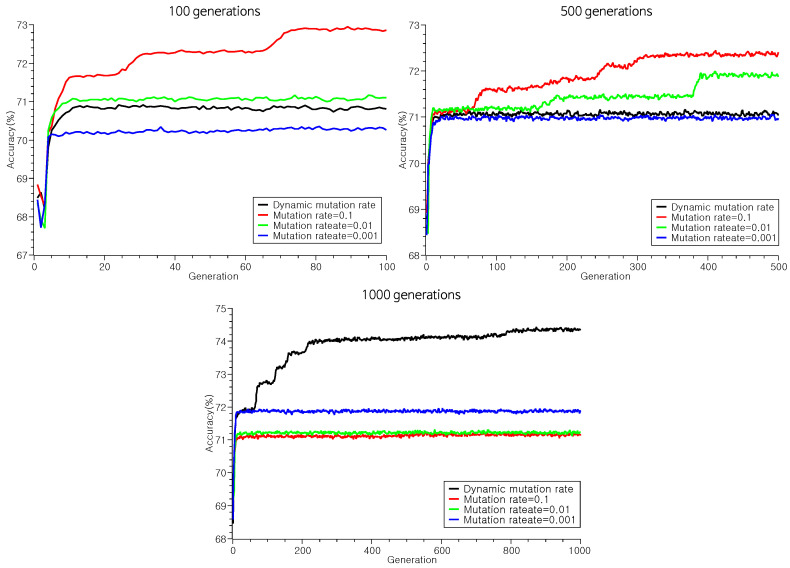
The average accuracy of the solutions founded by our genetic algorithm according to mutation rate.

Considering the convergence dynamics of solutions, the efficacy of dynamic HMCR in our experiment comes into question. Dynamic HMCR was implemented to avert stagnated convergence and procure more consistent solutions. However, as depicted in Figure 5, there is no notable alteration in the convergence patterns of the solution sets between dynamic and fixed HMCRs. The parity in convergence speed between dynamic and fixed HMCRs stems from the identical dynamic mutation rates applied. This showcases that dynamic parameter tuning only hastens convergence or improves performance when using 1000 generations (see Figure 6).

These findings suggest that while dynamic parameter tuning was proposed as an alternative to the fixed parameters, it does not inherently confer advantages and may yield a comparable performance to a method without dynamic tuning. Hence, introducing dynamic parameter tuning demands caution, and exploring alternative dynamic tuning approaches such as exponential scaling [32] or synchronizing with other parameters [55] is prudent.

### 4.3. Summarization

To wrap up the discussion, our experimental findings indicate that employing metaheuristic algorithms can enhance sarcopenia diagnosis performance by eliminating irrelevant factors. We have demonstrated that these methods are competitive with traditional feature selection techniques and can even outperform them under certain conditions. This suggests their potential applicability to machine learning for sarcopenia diagnosis. Additionally, we have discussed the importance of fine-tuning parameters and algorithm convergence in achieving successful outcomes, providing insights for future research directions.

However, despite these advancements, our study’s results have limitations concerning interpreting how the extracted features relate organically to sarcopenia diagnosis. Our study’s improved machine learning performance is attributed to the efficacy of metaheuristic algorithms and their reliance on black-box methods. Notably, metaheuristic-based feature selection with binary encoding lacks consideration of the importance of selected features, thereby reducing explanatory potential compared to traditional methodologies. This opacity and difficulty in explanation raise ethical concerns regarding applying our findings in clinical practice [56,57]. While efforts to introduce explainable AI aim to address these concerns [58], their impact on meaningful human decision-making still needs to be improved [59]. Moreover, interpretations provided by explainable AI may not sufficiently support meaningful decision-making at the patient level, necessitating improvements in evidence-based justifications [60]. It is crucial to consider uncertainties, clinician reasoning, and potential errors to utilize our results in real-world clinical practice effectively [56]. Further studies should focus on examining the outcomes of screened features to enhance the explanatory power of our findings and mitigate the challenges associated with AI interpretation.

We must also acknowledge the inherent limitations of metaheuristic algorithms themselves. While they excel in finding optimal solutions for NP-hard problems such as feature selection and offer many algorithms to explore, they have their drawbacks. According to the no-free lunch theorem, metaheuristics do not consistently maintain a state-of-the-art status and may become trapped in local optima. Moreover, their performance relies heavily on the computational methods used to reproduce solutions, and if there is significant overlap during the solution reproduction process, the algorithm may only consider a narrow range of feature sets [61]. Addressing these challenges will require further research involving the development of new metaheuristic approaches and experimentation under stricter constraints.

## 5. Conclusions

This study introduced and applied metaheuristic-based feature selection methods for diagnosing sarcopenia, primarily focusing on muscle strength data derived from survey data. Our experiments utilized genetic algorithms and harmony search, demonstrating their capacity to enhance machine learning performance in sarcopenia diagnosis. The findings indicate that genetic algorithms and harmony search contributed positively to machine learning performance. This was especially evident with the wrapper method algorithm design, which showcased competitive outcomes compared to alternative methodologies. Notably, the best results were observed using the support vector machine with harmony search and an HMCR set at 0.85, yielding an accuracy of 0.785 and a weighted F1 score of 0.782. These findings indicate that the metaheuristic approach is on par with other feature selection methods.

However, while these experiments highlight the potential of metaheuristic-based feature selection in improving sarcopenia diagnosis, they also unveil limitations. Notably, these methods need help identifying smaller feature sets. This suggests that metaheuristics may require fine-tuning parameters such as the number of generations and could benefit from regulations limiting feature set size. We also concluded that improper parameter dynamic tuning not only fails to enhance performance but can also potentially lead to performance degradation. Significantly, the methods in this study effectively enhanced machine learning performance. However, a limitation lies in the need for several explanatory pieces of evidence regarding how feature selection impacts machine learning for sarcopenia diagnosis.

In the future, applying metaheuristic-based feature selection beyond survey data to various dataset types in sarcopenia diagnosis holds promise. Furthermore, The findings of this study cannot be extrapolated to all surveys, as each survey serves a distinct purpose and targets a specific population. Therefore, further investigation is necessary to explore the applicability and effectiveness of feature selection methods across different survey contexts. Moreover, forthcoming research endeavors could incorporate SHAP (SHapley Additive exPlanations) [62] or employ explainable feature selection techniques [63] to address the explainability of machine learning processes and outcomes, a limitation of our current study. We expect that further refinement of the algorithms presented in this study and diversification of preprocessing methods will improve performance in subsequent studies.

## Figures and Tables

**Table 1:** Data distribution of sarcopenia and normal data.

Data	Sarcopenia	Normal	Total
Training set	2051	3139	5190
Test set	513	785	1298
Total	2564	3924	6488

**Table 2:** Genetic algorithm parameters in our experiment.

Parameter	Value
Population	30
Mutation rate	0.01
Parent selection rate	0.5

**Table 3:** Harmony search parameters in our experiment.

Parameter	Value
HMS	30
HMCR	0.85
PAR	0.7

**Table 4:** The ranges of dynamic parameters in this experiment.

Parameter	Value
${\mathrm{HMCR}}_{\min}$	0.7
${\mathrm{HMCR}}_{\max}$	1.0
$\mathrm{Mutation}\;{\mathrm{rate}}_{\min}$	0.001
$\mathrm{Mutation}\;{\mathrm{rate}}_{\max}$	0.1

**Table 5:** Machine learning performance on various feature sets. The data showcasing the highest performance for each machine learning algorithm are highlighted in bold for reference.

Dataset		J48			RandomForest			SMO			NaiveBayes			Selected Features	Time Costs(s)
		Accuracy	Weighted F1	AUC	Accuracy	Weighted F1	AUC	Accuracy	Weighted F1	AUC	Accuracy	Weighted F1	AUC		
GA	100	**0.728**	**0.722**	0.705	0.742	0.729	0.808	0.760	0.757	0.740	0.718	0.718	0.777	327.0	145.34
	500	0.722	0.715	0.692	0.733	0.717	0.794	0.752	0.748	0.729	0.722	0.717	0.770	181.0	469.01
	1000	0.700	0.683	0.674	0.729	0.708	0.800	0.742	0.737	0.717	0.715	0.713	0.761	277.2	951.81
HS	1000	**0.728**	**0.722**	0.705	**0.750**	0.736	0.822	0.773	0.770	0.752	0.743	0.738	0.803	250.0	207.67
	5000	**0.728**	**0.722**	**0.706**	0.748	0.733	**0.830**	0.784	0.781	0.764	**0.754**	**0.748**	**0.808**	238.0	848.32
	10,000	0.727	0.721	0.705	0.746	0.730	0.824	**0.785**	**0.782**	**0.765**	0.749	0.744	0.807	233.8	1560.57
CFS		0.705	0.695	**0.706**	0.728	0.721	0.781	0.731	0.720	0.694	0.713	0.714	0.780	18.0	3.82
IG		0.711	0.704	0.682	0.746	**0.737**	0.818	0.747	0.742	0.721	0.715	0.716	0.773	94.0	1.72
Original		**0.728**	0.721	**0.706**	0.743	0.728	0.813	0.760	0.760	0.750	0.701	0.704	0.773	778.0	-

**Table 6:** Performance comparison of harmony search with dynamic HMCR and fixed HMCR. The data showcasing the highest performance for each machine learning algorithm are highlighted in bold for reference.

Dataset		J48			RandomForest			SMO			NaiveBayes		
		Accuracy	Weighted F1	AUC	Accuracy	Weighted F1	AUC	Accuracy	Weighted F1	AUC	Accuracy	Weighted F1	AUC
Dynamic HMCR	100	0.727	0.720	0.705	0.753	0.739	0.822	0.768	0.765	0.748	0.740	0.736	0.795
	500	**0.729**	0.722	**0.707**	0.746	0.732	0.829	0.777	0.775	0.759	0.749	0.744	0.806
	1000	0.726	0.720	0.704	0.746	0.729	0.827	0.783	0.781	**0.766**	0.751	0.747	**0.808**
HMCR = 0.7	1000	**0.729**	**0.723**	**0.707**	0.746	0.734	0.815	0.782	0.777	0.758	0.740	0.734	0.797
	5000	0.728	0.722	0.705	0.748	0.735	0.823	0.780	0.776	0.711	0.747	0.741	0.805
	10,000	0.727	0.719	**0.707**	**0.754**	**0.743**	0.826	0.778	0.774	0.753	0.745	0.739	0.807
HMCR = 0.85	1000	0.728	0.722	0.705	0.750	0.736	0.822	0.773	0.770	0.752	0.743	0.738	0.803
	5000	0.728	0.722	0.706	0.748	0.733	**0.830**	0.784	0.781	0.764	**0.754**	**0.748**	**0.808**
	10,000	0.727	0.721	0.705	0.746	0.730	0.824	**0.785**	**0.782**	0.765	0.749	0.744	0.807

**Table 7:** Performance comparison of genetic algorithm with dynamic mutation rate and fixed parameters. The data showcasing the highest performance for each machine learning algorithm are highlighted in bold for reference.

Dataset		J48			RandomForest			SMO			NaiveBayes		
		Accuracy	Weighted F1	AUC	Accuracy	Weighted F1	AUC	Accuracy	Weighted F1	AUC	Accuracy	Weighted F1	AUC
Dynamic mutation rate	100	0.727	0.721	0.706	0.742	0.728	0.808	**0.774**	**0.771**	**0.753**	0.718	0.718	0.774
	500	**0.729**	**0.723**	**0.707**	**0.747**	**0.734**	**0.814**	0.749	0.747	0.732	0.714	0.715	0.775
	1000	0.726	0.719	0.703	0.744	0.730	0.803	0.760	0.756	0.737	0.717	0.717	0.777
Mutation rate = 0.1	100	0.721	0.712	0.693	0.733	0.719	0.800	0.769	0.765	0.745	0.720	0.718	0.777
	500	0.726	0.720	0.701	0.741	0.726	0.809	0.769	0.761	0.743	0.721	0.719	0.775
	1000	0.728	0.721	0.705	0.746	0.733	0.812	0.769	0.766	0.749	**0.729**	**0.727**	**0.779**
Mutation rate = 0.01	100	0.728	0.722	0.705	0.742	0.729	0.808	0.760	0.757	0.740	0.718	0.718	0.777
	500	0.722	0.715	0.692	0.733	0.717	0.794	0.752	0.748	0.729	0.722	0.717	0.770
	1000	0.700	0.683	0.674	0.729	0.708	0.800	0.742	0.737	0.717	0.715	0.713	0.761
Mutation rate = 0.001	100	0.727	0.721	0.703	0.743	0.728	0.808	0.759	0.757	0.741	0.714	0.715	0.771
	500	0.726	0.719	0.700	0.738	0.724	0.801	0.741	0.739	0.724	0.718	0.718	0.770
	1000	0.726	0.719	0.699	0.741	0.727	0.809	0.750	0.748	0.731	0.726	0.725	0.778

## Data Availability

KLoSA data used in this study can be obtained from https://survey.keis.or.kr/eng/klosa/klosa01.jsp (accessed on 3 November 2023).

## Abbreviations

The following abbreviations are used in this manuscript:

GA	genetic algorithm;
HS	harmony search;
CFS	CfsSubsetEval in WEKA;
IG	InfoGainAttributeEval in WEKA;
KLoSA	Korean Longitudinal Study of Aging.

## Appendix A. Parameter for Machine Learning

As noted in Section 3.3, we applied the default parameters offered by WEKA [46] for conducting the machine learning algorithms. The key parameters are outlined in Table A1.

**Table A1:** Key parameters of machine learning algorithm applied in this study.

Algorithm	Parameter
J48	-C 0.25 -M 2
RandomForest	-K 0 -M 1.0 -V 0.001 -S 1
SMO	-C 1.0 -L 0.001 -P 1.0E-12 -K PolyKernel -calibrator Logistic
NaiveBayes	Do not have specific parameter
